# Disproportionate Mediastinal Shift From an Isolated Right Middle Lobe Collapse: A Case Report

**DOI:** 10.7759/cureus.106924

**Published:** 2026-04-12

**Authors:** Sarib Sultan, Yasmin Obeidi, Mohammad Mahdi, Osamah Salim, Sarim Sultan, Adeel Ahmed, Mario Loomis

**Affiliations:** 1 Department of Research, Sam Houston State University College of Osteopathic Medicine, Conroe, USA; 2 Department of Research, Lincoln Memorial University DeBusk College of Osteopathic Medicine, Knoxville, USA; 3 Department of Clinical Anatomy, Sam Houston State University College of Osteopathic Medicine, Conroe, USA

**Keywords:** cardiac dextroposition, copd patients, mediastinal shift, pulmonary atelectasis, right middle lobe, right middle lode collapse

## Abstract

Mediastinal displacement toward the side of lung volume loss is a well-recognized radiographic feature of atelectasis. Significant mediastinal shift most commonly occurs when larger lung segments, such as the right upper or lower lobes, collapse. Because the right middle lobe (RML) contributes a relatively small proportion of total lung volume, isolated RML collapse typically produces minimal mediastinal deviation.

The case in this report is of an 83-year-old woman with chronic obstructive pulmonary disease (COPD) and congestive heart failure (CHF), who presented with progressive dyspnea and increased oxygen requirements. Chest radiography demonstrated opacification of the right middle lobe with marked rightward displacement of the cardiac silhouette. Computed tomography (CT) of the chest confirmed isolated collapse of the right middle lobe with rightward mediastinal and cardiac displacement, without involvement of the right upper or lower lobes. Bronchoscopy revealed narrowing of the right middle lobe bronchus due to floppy cartilage consistent with bronchomalacia and no evidence of endobronchial mass or malignancy. The patient improved with conservative medical management, including diuresis, bronchodilators, and pulmonary hygiene.

This case demonstrates that isolated RML collapse can produce significant mediastinal and cardiac displacement. Clinicians should recognize that even the collapse of relatively small lung segments may generate substantial radiographic shift, particularly in patients with underlying airway structural abnormalities or pulmonary hyperinflation.

## Introduction

Right middle lobe (RML) collapse is a recognized clinical entity frequently associated with airway obstruction, mucus plugging, inflammatory airway disease, or structural bronchial abnormalities commonly referred to as middle lobe syndrome [1]. Radiographic findings commonly include triangular opacification of the RML, obscuration of the right heart border, and localized volume loss [1].

Mediastinal displacement toward the side of lung volume loss is a well-established radiographic sign of atelectasis [2]. The degree of mediastinal shift generally correlates with the volume of lung involved, with collapse of larger lung segments such as the right upper or right lower lobes more commonly producing clinically apparent mediastinal deviation [2].

Because the RML contributes a relatively small portion of total lung volume, isolated RML collapse typically produces minimal mediastinal displacement and may be radiographically subtle [1]. Reports describing significant mediastinal shift resulting solely from isolated RML collapse remain limited.

We present a case of isolated RML collapse producing marked rightward cardiac and mediastinal displacement in a patient with COPD, highlighting the potential for disproportionate mediastinal shift even with collapse of relatively small lung segments.

## Case presentation

An 83-year-old female patient with a medical history significant for chronic obstructive pulmonary disease (COPD), congestive heart failure, lymphedema, and type 2 diabetes mellitus presented with progressive dyspnea, orthopnea, and productive cough. She reported increased oxygen requirements and recent nonadherence to prescribed diuretic therapy. She was a former smoker with an unknown pack-year smoking history. 

On presentation, the patient was tachypneic with oxygen saturation of 92% while receiving 4 L of oxygen via nasal cannula. Lung examination revealed decreased breath sounds in the right mid-lung field with bibasilar crackles (Table 1).

**Table 1:** Vital signs on presentation

Vital Sign	Patient's value	Reference Range
Blood pressure (systolic/diastolic; mmHg)	172 / 72	<120 / <80
Respiratory rate (breaths/min)	20	12–20
Oxygen saturation (SpO₂; %room air)	92 (on 4 liters nasal cannula)	≥95

Laboratory studies demonstrated an elevated brain natriuretic peptide (BNP) level of 2179 pg/mL. Venous blood gas revealed compensated hypercapnia with a pCO2 of 62 mmHg and normal pH (Table 2).

**Table 2:** Laboratory values on presentation Abbreviations: BNP: B-type natriuretic peptide; pCO₂: partial pressure of carbon dioxide; HCO₃⁻: bicarbonate; SpO₂: peripheral oxygen saturation.

Parameter	Value	Reference range
B-type natriuretic peptide (BNP; pg/mL)	2179	<100
Venous blood gas pH	7.38	7.31–7.41
Venous partial pressure of carbon dioxide (pCO₂; mmHg)	62	41–51
Bicarbonate (HCO₃⁻; mmol/L)	36.7	22–28

**Figure 1:**
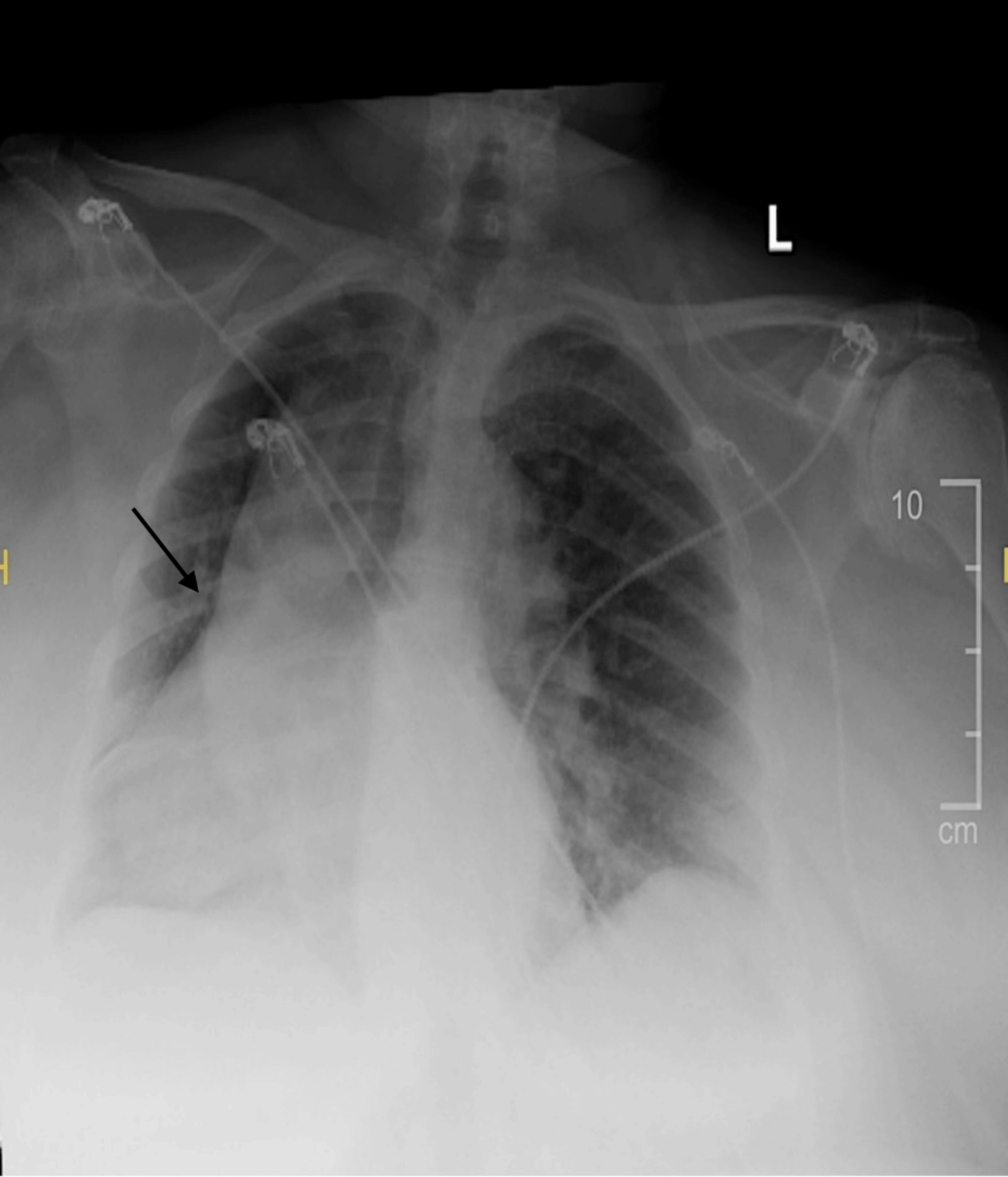
Chest radiograph demonstrating right middle lobe collapse with rightward displacement of the cardiac silhouette Black arrow indicates the region of right middle lobe atelectatic opacity/volume loss.

Chest radiography demonstrated opacification and volume loss involving the RML with marked rightward displacement of the cardiac silhouette (Figure 1).

Despite the apparent rightward cardiac position, the cardiac apex remained oriented toward the left, and the gastric bubble was visualized on the left side, excluding congenital dextrocardia. Prior chest imaging was not available for comparison. 

**Figure 2:**
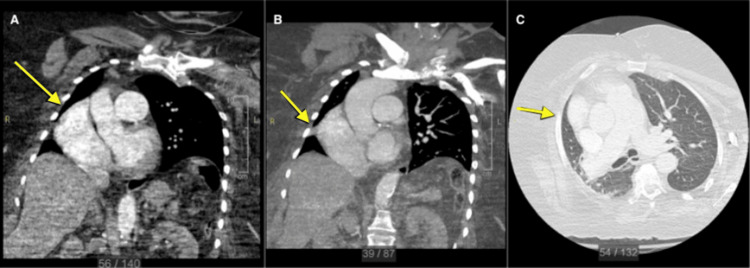
CT chest demonstrating isolated right middle lobe (RML) collapse and mediastinal displacement (A) Coronal CT demonstrating mediastinal shift, with the yellow arrow indicating rightward mediastinal displacement; (B) Coronal CT showing fissure displacement and RML volume loss, with the yellow arrow indicating the displaced fissure/RML volume loss; (C) Axial CT demonstrating triangular RML atelectasis, with the yellow arrow indicating the triangular atelectatic RML opacity.

Computed tomography (CT) of the chest confirmed isolated collapse of the RML with associated rightward mediastinal and cardiac displacement. The right upper and right lower lobes remained aerated without evidence of additional lobar collapse. CT imaging demonstrated a triangular opacity between the minor and major fissures consistent with RML atelectasis (Figure 2).

Diagnostic bronchoscopy was performed to evaluate for possible airway obstruction. It revealed partial narrowing of the right middle lobe bronchus due to floppy cartilage consistent with bronchomalacia. No endobronchial masses or lesions were identified. Bronchoalveolar lavage was performed. Cultures demonstrated rare white blood cells and rare gram-positive cocci with growth of normal respiratory flora without evidence of pathogenic organisms. Acid-fast bacillus (AFB) smear and culture were negative, with no acid-fast bacilli isolated after six weeks of incubation. Fungal smear using KOH/Calcofluor preparation demonstrated no fungal elements, and fungal cultures showed no yeast or mold growth after four weeks.

During hospitalization, the patient was managed for concurrent acute on chronic COPD and heart failure exacerbations. She was treated with intravenous furosemide for volume overload in the setting of elevated BNP, along with bronchodilator therapy (nebulized albuterol-ipratropium), inhaled corticosteroids, and systemic corticosteroids for COPD exacerbation. Her oxygen requirements improved from 4 L nasal cannula on admission to 3 L by discharge. These interventions likely improved ventilation and reduced airway inflammation, contributing to resolution of symptoms. The patient’s respiratory status improved with conservative management, and no invasive intervention was required.

## Discussion

Mediastinal displacement toward the side of lung volume loss is a well-established radiographic feature of atelectasis. The degree of displacement generally correlates with the volume of lung involved, with collapse of larger lobes such as the right upper or right lower lobes more commonly producing clinically apparent mediastinal shift [2,3]. In contrast, the RML contributes a relatively small proportion of total lung volume, and isolated RML collapse is typically associated with minimal or subtle mediastinal deviation [1]. In many cases, RML collapse manifests as loss of the right heart border on chest radiography, a classic example of the silhouette sign [3].

Given this, a significant mediastinal shift would not typically be expected in an isolated RML collapse. Prior literature on RML syndrome and lobar atelectasis consistently describes subtle radiographic findings, most commonly obscuration of the right heart border and localized volume loss rather than marked mediastinal displacement [1,3]. By contrast, clinically apparent mediastinal shift is more often associated with collapse of larger lobes, particularly the right upper or lower lobes, where the degree of displacement correlates with the volume of lung involved [2]. In this context, the marked rightward mediastinal and cardiac displacement seen in our patient appears disproportionate to the expected volume loss from isolated RML atelectasis and therefore represents an unusual radiographic presentation.

It also suggests that additional factors beyond simple volume loss may significantly influence mediastinal positioning. Several factors likely contributed to the exaggerated mediastinal shift observed in this patient. Bronchoscopy demonstrated bronchomalacia involving the RML bronchus, which may have promoted dynamic airway collapse, impaired ventilation, and persistent lobar volume loss. Bronchomalacia has been associated with airway instability and recurrent atelectasis due to increased collapsibility during expiration [4]. In addition, underlying COPD likely played a significant role. COPD-related hyperinflation of the remaining lung parenchyma may amplify the mechanical effects of localized volume loss by increasing expansion of adjacent lung segments [5,6]. This combination of localized collapse and compensatory hyperinflation can exaggerate mediastinal displacement beyond what would be expected based on lobar volume alone. Fissure displacement is a recognized radiologic sign of atelectasis and supports that the observed mediastinal shift was secondary to true lobar volume loss rather than compensatory hyperinflation alone [7]. 

Heart failure likely also contributed to the patient’s clinical presentation. On admission, she had a markedly elevated BNP of 2179 pg/mL, bibasilar crackles on examination, recent nonadherence to diuretic therapy, and symptomatic improvement following intravenous diuresis. These findings support concurrent volume overload as a contributor to dyspnea and hypoxemia. Although heart failure alone would not explain the focal fissure displacement and isolated RML atelectasis seen on CT, pulmonary vascular congestion and interstitial/alveolar fluid may have worsened respiratory mechanics and reduced physiologic reserve, thereby amplifying the clinical impact of the lobar collapse.

These findings support a mechanistic explanation in which disproportionate mediastinal displacement arises from the interaction between localized lobar collapse and altered lung mechanics, rather than from volume loss alone. They also highlight an important consideration in radiographic interpretation. Significant mediastinal displacement is often attributed to collapse of larger lung segments or mass effect; however, this case demonstrates that even collapse of smaller lobes such as the RML can produce substantial mediastinal shift when compounded by airway instability and altered lung mechanics [8]. Recognition of this phenomenon is important to avoid misinterpretation of imaging findings as large lobar collapse, mass effect, or alternative pathology, particularly in patients with underlying pulmonary disease.

This report contributes to existing literature by demonstrating that mediastinal displacement in atelectasis is not solely dependent on the size of the affected lobe but may be significantly influenced by underlying pulmonary conditions such as bronchomalacia and COPD-related hyperinflation. Further characterization of these contributing factors may improve understanding of variability in radiographic presentations of lobar collapse.

## Conclusions

Isolated RML collapse can produce significant mediastinal and cardiac displacement despite the relatively small volume of the lobe. In this case, structural airway abnormality in the form of bronchomalacia, along with underlying COPD, likely contributed to persistent lobar collapse and exaggerated mediastinal shift. This highlights that radiographic findings may not always correlate linearly with the expected volume of lung involvement. 

This case emphasizes the importance of considering underlying airway pathology and altered pulmonary mechanics when interpreting disproportionate mediastinal displacement on imaging. Recognition that small lobar collapse, particularly in the presence of conditions such as COPD and bronchomalacia, can result in marked radiographic shift may help prevent misdiagnosis of larger lobar collapse, mass effect, or other intrathoracic pathology. Increased awareness of this phenomenon can improve diagnostic accuracy and guide appropriate clinical management, particularly in patients with complex pulmonary comorbidities.
